# From code to care in hours: Why “go fast and fix things” is the new medical imperative

**DOI:** 10.1002/jeo2.70768

**Published:** 2026-05-15

**Authors:** Oriol Pujol, Felix C. Oettl, Jacob F. Oeding, James A. Pruneski, Balint Zsidai, Stefano Zaffagnini, Kristian Samuelsson

**Affiliations:** ^1^ Knee Surgery Unit, Orthopaedic Surgery Department Vall d'Hebron University Hospital, Universitat Autònoma de Barcelona (Departament de Cirurgia) Barcelona Spain; ^2^ Knee Surgery Unit, iMove Traumatology Barcelona Spain; ^3^ Orthopaedic Surgery Department Balgrist University Hospital, University of Zürich Zurich Switzerland; ^4^ Department of Orthopaedics, Institute of Clinical Sciences, Sahlgrenska Academy University of Gothenburg Gothenburg Sweden; ^5^ Department of Orthopaedic Surgery, Tripler Army Medical Center Honolulu Hawaii USA; ^6^ Sahlgrenska Sports Medicine Center Gothenburg Sweden; ^7^ Orthopaedic Surgery Department, Institute of Clinical Sciences, Sahlgrenska Academy University of Gothenburg Gothenburg Sweden; ^8^ Orthopaedic Surgery Department Skåne University Hospital Malmö/Lund Sweden; ^9^ Clinica Ortopedica e Traumatologica II, IRCCS Istituto Ortopedico Rizzoli Bologna Italy; ^10^ Department for Orthopaedics Sahlgrenska University Hospital Mölndal Sweden

**Keywords:** artificial Intelligence, digital health, machine learning, target product profile, validation

## Abstract

Recent technological advances in artificial intelligence and machine learning have led to rapid changes in clinical informatics. With the release of new models, the barrier to creating medical software has markedly decreased. As noted in the now‐viral article by Matt Shumer, we have moved from a world where building a clinical application took a team of engineers working over the course of months to years, to one where a single clinician can develop and deploy a working prototype in hours to days. However, the same innovation driving this ‘explosion’ of tools also carries the risk of flooding health systems with unvalidated ‘AI slop.’ As prohibiting tools developed through AI runs the risk of stifling innovation and limiting potentially impactful discoveries, we suggest abandoning the slow, fear‐based resistance to AI adoption. Further, we propose a ‘go fast and fix things’ framework—utilising target product profiles, regulatory sandboxes, and continuous audit—to harness the speed of generation and prototyping while rigorously validating clinical utility.

**Abbreviations**
AI: artificial intelligence
LLM: large language model
TPP: target Product Profile

## THE SHIFT—‘EASE OF CREATION’

For the last decade, the primary bottleneck in digital health was engineering bandwidth. If a department wanted an artificial intelligence (AI) tool to automate procedures, such as patient scheduling or triaging lab results, they needed budget, developers and time. That era has ended. As Matt Shumer's widely read analysis highlighted [7], the new class of models (GPT‐5.3 Codex, Claude Code or Google Antigravity) no longer just writes code fragments. Foundation models, which can be broadly defined as AI systems pre‐trained on vast and diverse datasets that can be adapted through prompting or fine‐tuning to perform a wide range of tasks—including code generation, language understanding and clinical decision support—, are able to build functional architectures based on natural language prompts. As a result, we are now witnessing the commoditization of the ‘builder’ role.

Improvements in large language models (LLMs) have made it possible to create more versatile AI tools, with the trajectory of OpenClaw being a recent example [1]. What began as a modest open‐source project for agentic secretarial workflows, such as managing repetitive tasks like referral sorting and ambient documentation, exploded in capability and adoption so rapidly that it was acquired by OpenAI in February 2026, just 3 months after its release. OpenClaw demonstrated that ‘secretarial’ AI is no longer a research project; it is a utility.

The implication for hospitals is profound. We are no longer constrained by *how* to build, only by *what* we should build. Clinicians now have the capacity to develop solutions to the problems they encounter each day by themselves, rather than waiting on others to build the solutions for them.

## THE VALIDATION TRAP

Importantly, ease of creation is not synonymous with safety of use. As the engineering barrier falls, the validation burden rises exponentially. We face a crisis similar to the one currently plaguing computer science, where ‘AI slop’—plausible‐sounding but scientifically vacuous content—is overwhelming peer review systems with low‐quality or even fake investigations [4]. In medicine, controlling ‘slop’ is not just an administrative burden; it has significant ramifications on patient care. Similar to the AI Act principles [6], we must stratify our validation approach based on risk [8]:

1. **Low‐risk (administrative):** Tools that summarise meetings, draft denial appeals or sort emails.
2. **High‐risk (diagnostic/therapeutic):** Tools that predict sepsis, flag complications or recommend drug dosages.

Low‐risk tools can be fast‐tracked, as errors may be inconvenient but are unlikely to result in fatality. However, high‐risk tools should undergo rigorous evaluation and validation due to their potential to cause significant patient harm.

The trap, as criticised by a recent *NEJM AI* article, is that now it is just as easy to build a sepsis predictor as it is to build a meeting scheduler [4]. But a sepsis predictor trained on biased data or deployed without workflow consideration has much greater clinical ramifications. We cannot allow the ‘ease of creation’ to bypass the rigour of clinical evidence. If we do, we risk a ‘validation debt’ that will bankrupt trust in digital health.

## THE PLAYBOOK: ‘GO FAST AND FIX THINGS’

Popularised in the technology sector, most notably by Facebook (now Meta Platforms) during its early growth phase in the 2010s, the phrase ‘move fast and break things’ is unacceptable in medicine. While this philosophy of rapid experimentation that prioritises speed and iteration over stability, accepting short‐term failures at the cost of innovation is clearly not suited for health‐related tools, ‘go slow and wait for perfect’ could also be considered unethical when patients are affected by inefficiencies. The crucial question at the heart of this evolution is how to balance innovation, ethics and safety [3].

**Figure 1:**
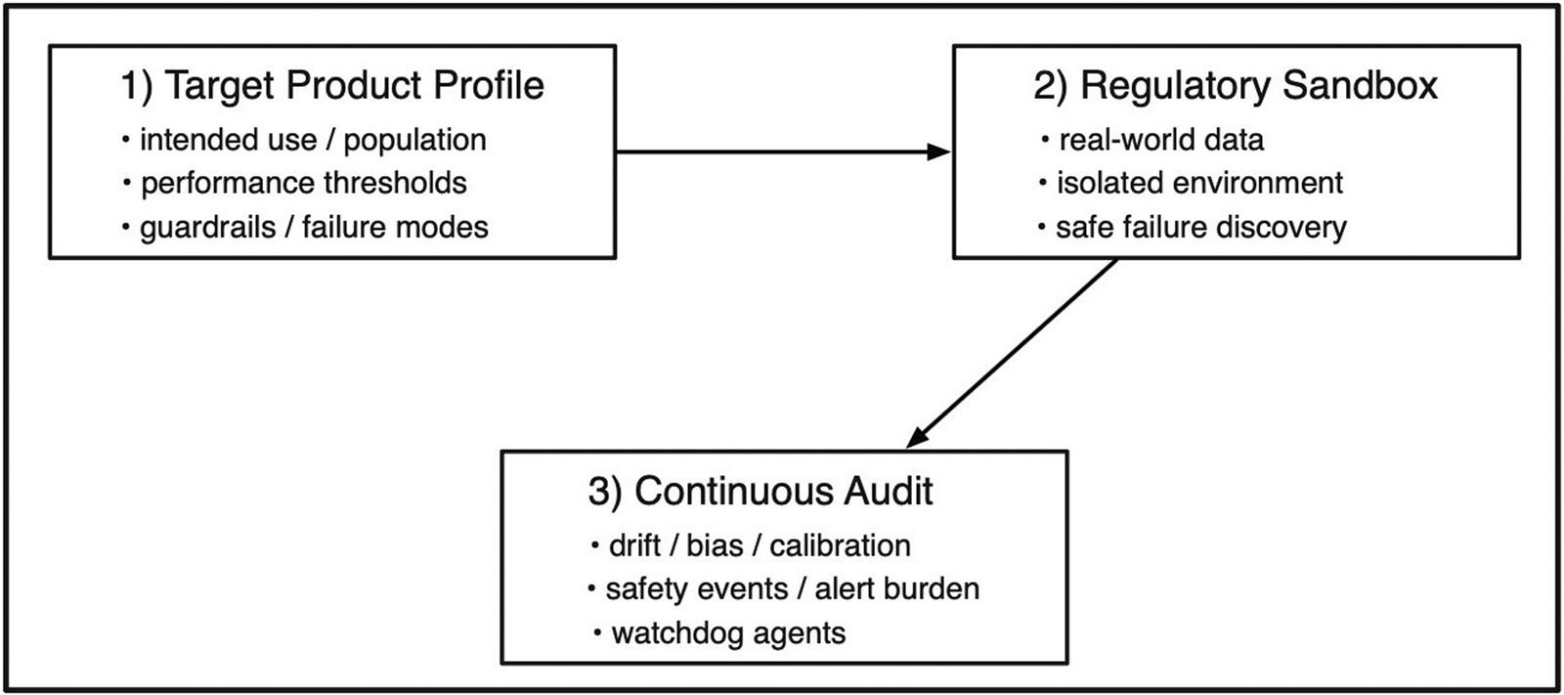
A ‘go fast and fix things’ framework preserves rapid iteration while enforcing clinical rigour. Target product profiles define the clinical specification up front, sandboxes enable safe real‐world testing, and continuous audit converts validation from a one‐time gate into an ongoing system.

We propose a ‘go fast and fix things’ methodology, integrating three core components (Figure 1):

1. **The blueprint before the build (target product profile):** Before a single prompt is sent to a coding engine to build an AI tool, the clinical utility must be defined. As proposed by Parsa et al., we must use Health AI target product profiles (TPPs) [5]. Health AI TTPs are structured, pre‐specified development blueprints that define an AI system intended clinical use, target population, required performance thresholds (e.g., sensitivity, specificity, calibration), workflow integration constraints, safety guardrails and acceptable failure modes before model development begins. These profiles help to ensure that technical optimisation is anchored to predefined clinical utility rather than post hoc performance adjustment. In other words, the AI builds to the specification; the specification does not change to fit the AI. This prevents the development of seemingly impressive tools with low clinical utility.
2. **Regulatory sandboxes (The TEMPO model):** Institutions should mimic the FDA's new TEMPO pilot and create internal ‘AI Sandboxes’ [2]. Sandboxes are safe and controlled environments where testing can be conducted to understand the opportunities and risks associated with a specific innovation. In these isolated clinical environments, AI tools can be deployed on real‐world data without full integration into the electronic health record [2]. This allows for the iterative ‘fixing’ of models in days, not years, observing failure modes safely before formal deployment.
3. **Continuous audit, not one‐time approval:** Validation is no longer a gate; it is a loop. Because these models are dynamic and the workflows they inhabit change, we need automated ‘watchdog’ agents, specialised models whose only job is to audit the outputs of the clinical models.

## CONCLUSION

Technological advances now allow the clinicians the opportunity to be more involved in digital health innovations. As the technical barriers to building AI tools fall, the need for proportionate, risk‐based validation rises. Most importantly, we must not let ease of development bypass clinical rigour. Archaic validation methods may promote the development of enormous amounts of unverified tools. But if we adopt a ‘go fast and fix things’ mentality—marrying the speed of generative coding with the rigour of sandboxed validation and TPPs—a new era of improved efficiency in healthcare may soon follow.

## CONFLICT OF INTEREST STATEMENT

Stefano Zaffagnini is editor‐in‐chief of *Journal of Experimental Orthopaedics* (JEO). Kristian Samuelsson is a member of the Board of Directors of Getinge AB (publ) and medtech advisor to Carl Bennet AB. The remaining authors declare no conflicts of interest.

## ETHICS STATEMENT

The authors have nothing to report.
